# An e‐Reporting Tool for Facilitating Submission of Veterinary Adverse Drug Reaction Reports

**DOI:** 10.1111/jvim.70173

**Published:** 2025-07-12

**Authors:** Heather Davies, Steven Smyth, Gina Pinchbeck, Munir Pirmohamed, Roy Savory, P. J. Noble, David Killick

**Affiliations:** ^1^ Institute of Infection, Veterinary and Ecological Sciences University of Liverpool Liverpool UK; ^2^ Institute of Systems Molecular and Integrative Biology University of Liverpool Liverpool UK; ^3^ Pharmacovigilance Unit Veterinary Medicines Directorate Addlestone UK

**Keywords:** adverse drug reaction, electronic health records, informatics, pharmacovigilance, veterinary medicine

## Abstract

**Background:**

Adverse events (AEs) are under‐reported in veterinary medicine. The ability to report AEs directly from the practice management system (PMS) has been suggested to facilitate reporting. The Small Animal Veterinary Surveillance Network (SAVSNET) informatics system provides an opportunity to integrate reporting into the workflow such that reports can be submitted directly to the National Competent Authority, the Veterinary Medicines Directorate (VMD).

**Objectives:**

1. Develop an AE reporting form linked to the PMS allowing for pre‐population of some fields from the electronic health record (EHR).
2. Analyze the quality of submitted reports.

**Animals:**

Animals attending United Kingdom (UK) first‐opinion veterinary practices participating in SAVSNET.

**Methods:**

An AE “reporting button” was developed and available in the normal clinical workflow for SAVSNET enrolled practices using the Robovet PMS. The button facilitated capture of pertinent information relating to AEs, including the ability to append clinical notes from the associated EHR. After submission, reports were automatically submitted daily to the VMD. Report quality was assessed using an adapted version of the vigiGrade scoring system, which was used to compare the quality of reports submitted to the VMD via standard routes to those submitted via SAVSNET. Assessment of reports submitted via SAVSNET, was conducted twice. First, considering only information contained in the report and second, considering information contained in both the report and associated clinical notes.

**Results:**

Sixty reports were submitted during the first 18 months by 42 different veterinary practices. The quality of SAVSNET reports was significantly improved by information contained within the clinical notes. These reports were more likely to be well‐documented than those submitted via standard routes.

**Conclusions and Clinical Importance:**

Adverse event reports populated using EHR data are well documented and can support efficient reporting of AEs in veterinary medicine.

**Abbreviations**
AE: adverse event
EHR: electronic health record
EMA: European Medicines Agency
MAH: marketing authorization holder
PMS: practice management system
SAVSNET: Small Animal Veterinary Surveillance Network
SPC: summary of product characteristics
VeDDRA: Veterinary Dictionary for Drug Regulatory Activities
VGVP: veterinary good pharmacovigilance practice
VMD: Veterinary Medicines Directorate

## 1 Introduction

Spontaneous reporting of adverse events (AEs) to national competent authorities and pharmaceutical companies by veterinary professionals and members of the public is central to post‐marketing pharmacovigilance. The Veterinary Medicines Directorate (VMD) is the national competent authority, also known as the regulatory body, responsible for ensuring the safety of veterinary medicinal products in the United Kingdom (UK). The VMD receives AE reports from veterinary professionals and members of the public, as well as from pharmaceutical companies (so‐called marketing authorization holders). Marketing authorization holders have a legal obligation to report AEs to national competent authorities (such as the VMD). Veterinary professionals are under no such obligation, but are instead encouraged to do so by the Royal College of Veterinary Surgeons (RCVS) Code of Professional Conduct [1, 2]. The VMD received 6139 AE reports in 2020 and used this information to update the product information for 88 products [3]. Despite the importance of AE reporting for the identification of emerging drug safety issues and further characterization of known AEs, evidence suggests substantial under‐reporting [4, 5, 6, 7, 8, 9] and therefore methods to facilitate reporting are of great interest.

In a recent survey of UK veterinary professionals, the ability to report AEs via the practice management system (PMS) was proposed as a facilitator of AE reporting [10]. Similar findings have been identified in surveys conducted in other locations [4, 5], thus indicating a promising avenue for investigation.

The Small Animal Veterinary Surveillance Network (SAVSNET) collects electronic health records (EHRs) from first‐opinion veterinary practices in the United Kingdom. A full description of SAVSNET data collection is provided elsewhere [11], but part of the process is a window that appears after each consultation requiring the veterinary professional to indicate the main presenting complaint for the consultation. This window is maintained by SAVSNET and compatibility testing between SAVSNET and the PMS is carried out before practices enroll in the project. As the main presenting complaint window appears after every consultation, it provides an opportunity to integrate a reporting functionality into the everyday clinical workflow.

Therefore, our main aim was to design and evaluate a PMS‐to‐VMD AE reporting functionality for integration into the SAVSNET main presenting complaint window. Doing so would allow for pre‐population of some data fields and data collation from the patients EHR, thus providing veterinary professionals with a more efficient direct reporting route to the VMD compared to current methods (i.e., web form or paper‐based reporting).

The specific objectives were: (1) to develop an AE reporting form linked to the PMS, allowing for pre‐population of some elements of the report from information contained in the EHR and delivery of the reports to the national competent authority (VMD) in an automated manner; and (2) to analyze the completeness and quality of submitted AE reports.

## 2 Materials and Methods

### 2.1 Report Form Design

**FIGURE 1:**
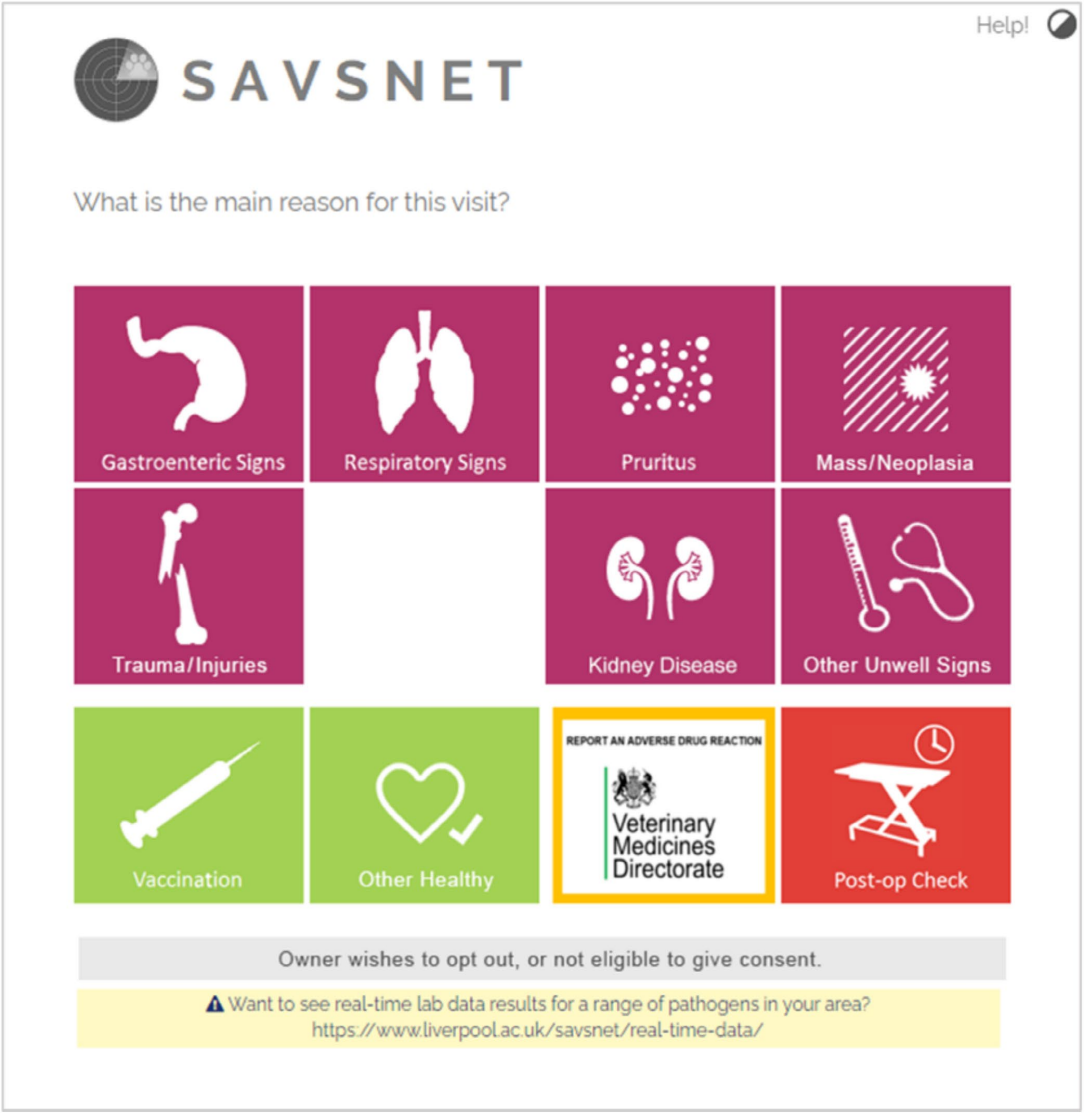
The AE reporting button was included on the SAVSNET main presenting complaint (MPC) window which is shown to participating veterinary practices following each consultation and practitioners must select the reason for which the animal was presented for care.

An AE report form was developed in collaboration with the VMD and, in order to complement existing reporting systems, fields were based on those in the VMD's online report form (https://www.gov.uk/report‐veterinary‐medicine‐problem/animal‐reacts‐medicine). The form was opened by selecting the “Report an Adverse Drug Reaction” tile on the SAVSNET main presenting complaint window (Figure 1). The term “adverse drug reaction” was chosen rather than AE to avoid confusion with the reporting of medication errors or near misses as part of existing clinical governance processes.

Information collection was separated into four sections, each shown on sequential screens: animal information, drug information, reaction information and contact information. The information captured on each screen, including details of the input and field type, is shown in Table 1. The guideline on veterinary good pharmacovigilance practices (VGVP) module “collection and recording of suspected adverse events for veterinary medicinal products” states that four minimum reporting criteria are required for an AE to be considered valid [12]. Of these criteria, two were captured automatically (patient and suspect product), and a third (reporter) was considered present by the VMD because of the report being associated with a unique practice identifier within the SAVSNET database. Consequently, reporters were only required to input a free text description of the AE into the “reaction description” field to create a valid report.

**TABLE 1:** Fields on the animal, drug, reaction and contact information screens of the AE reporting form, including details of the input types.

	Field name	Input type	Field type	Requirement
Animal	Species	Automated	N/A	Non‐mandatory
	Breed	Automated	N/A	Non‐mandatory
	Date of birth	Automated	N/A	Non‐mandatory
	Sex	Automated	N/A	Non‐mandatory
	Neuter status	Automated	N/A	Non‐mandatory
	Weight	Manual	Numeric	Non‐mandatory
Drug(s)	Suspect drug	Automated	Drop down (recently dispensed products) with optional free‐text	Mandatory
	Marketing authorization number	Manual	Free‐text	Non‐mandatory
	Batch number	Manual	Free‐text	Non‐mandatory
	Route of administration	Manual	Drop down (standardized list)	Non‐mandatory
	Drug start date	Manual	Date picker	Mandatory
	Duration of administration	Manual	Numeric	Non‐mandatory
	Duration of administration (units)	Manual	Drop down (hours, days, weeks, months)	Non‐mandatory
	Dosage details	Manual	Free‐text	Non‐mandatory
	Occupation of administrator	Manual	Drop down (vet, vet nurse, owner, or other non‐veterinary professional)	Non‐mandatory
	Concurrent products	Automated	Free‐text	Non‐mandatory
Reaction	Date of reaction	Manual	Date picker	Mandatory
	Duration of reaction	Manual	Numeric	Non‐mandatory
	Duration of reaction (units)	Manual	Drop down (hours, days, weeks, months)	Non‐mandatory
	Was the reaction fatal?	Manual	Drop down (yes, no)	Non‐mandatory
	Details on other animals treated	Manual	Free‐text	Non‐mandatory
	Reaction description	Manual	Free‐text	Mandatory
	Append clinical notes?	Automated	Tick box to select then clinical notes automatically appended	Non‐mandatory
	Submitted to the manufacturer?	Manual	Drop down (yes, no)	Mandatory
Contact	Practice postcode area	Automated	Free‐text (to allow removal)	Non‐mandatory
	Reporter contact details	Manual	Free‐text	Non‐mandatory
	Reporter email address	Manual	Free‐text	Non‐mandatory
	Reporter telephone number	Manual	Free‐text	Non‐mandatory
	Wish to receive a copy via email?	Manual	Drop down (yes, no)	Mandatory

Fields capturing information regarding suspect and concurrent drugs were pre‐populated with recently sold products. However, additional products could be added in free text to account for products dispensed or purchased elsewhere. Products also could be removed, which was important given that some non‐pharmaceutical items (e.g., “puppy check”) could appear in these fields because of the nature of invoice and billing systems at individual practices.

The reaction description was captured in a mandatory free text field. In recognition of the fact that the free text clinical notes contained within the EHR may provide useful context to the report, the option to append the last two months of anonymized clinical notes was provided to reporters. As veterinary professionals may have provided a thorough description or other important information (including treatments) in the clinical notes relating to the current consultation, this field was later updated such that the anonymized clinical notes from the current consultation were automatically appended after report submission. After this change, the ability to append clinical notes from the previous two months was retained.

After all fields were completed, reporters were directed to the submission screen which contained a tick box consent statement. After selecting “submit,” a reference number was provided which reporters could copy and paste into the clinical notes for record‐keeping purposes, if desired. The European Medicines Agency (EMA) guideline on data elements for electronic submission [13] was used to create an XML schema to enable direct gateway submission to VMD which occurred at 02:00 on a daily basis.

### 2.2 Ethical Approval

The SAVSNET project has existing ethical approval from the University of Liverpool Research Ethics Committee (RETH001081). An amendment for incorporating the AE reporting button was submitted and approved before the launch of the pilot study.

### 2.3 Pilot Study

A demonstration of reporting an AE using the SAVSNET reporting button was provided to members of the SAVSNET team and to the pharmacovigilance department at the VMD. Directly after the demonstration, VMD provided feedback to the research team including suggestions regarding comprehension and ease of use, and updates were made as appropriate. All SAVSNET practices then were invited via email to take part in a pilot study. Practices that agreed to take part in the pilot study were asked to submit five test cases and complete a short feedback form.

### 2.4 Launch

The reporting button was rolled out to all SAVSNET practices using the Robovet PMS on 18‐Sep‐2020. The launch was advertised widely with practices sent an information pack by email, an announcement placed on the SAVSNET dashboard, a letter published in the Veterinary Record, and additional announcements made in the veterinary press (Vet Times and British Small Animal Veterinary Association [BSAVA] Companion).

### 2.5 Report Assessment

For each AE report event “expectedness,” suspect product type and event description were classified by one author, who has experience in AE intake and reporting. Adverse events were considered to be ‘expected’ if the event was listed in the most recent version of the Summary of Product Characteristics (SPC). Products listed as the suspect product on the reports were classified using the ATCVet code first level groups, identified either from the SPC or by searching the ATCVet code index by active substance (https://atcddd.fhi.no/atcvet/). Descriptions of AEs were coded using the Veterinary Dictionary for Drug Regulatory Activities (VeDDRA) low level terms (LLTs) [14]. Descriptive statistics were calculated using Microsoft Excel (2016).

Report completeness was assessed using a tool adapted from the World Health Organization (WHO) vigiGrade completeness score criteria [15]. In this assessment, the individual elements of a report are weighted according to their relative importance in causality assessment. Although the original vigiGrade scoring system is automated, the adapted criteria were employed manually in our study. The scoring tool consisted of 10 separate elements, with a score of one assigned if the element was present or a penalty applied for missing or incomplete information. All scoring was conducted independently by two reviewers (a veterinary surgeon practicing in an academic referral center with additional experience in drug safety and a postgraduate researcher with experience in pharmacovigilance) and discrepancies were resolved by discussion to reach consensus. The final report score was calculated by multiplying together the scores for each individual element. As for the original vigiGrade tool, reports were considered well‐documented if they achieved a score of 0.8 or higher [15]. The elements assessed and penalties applied are shown in Table 2.

**TABLE 2:** Scoring criteria used to assess the completeness of reports submitted via the SAVSNET AE reporting button, adapted from VigiGrade [13].

Element	Relevant field(s)	Scoring
Time‐to‐onset	StartDateDrug StartDateReaction [ReactionDesc]	If both fields populated and drug precedes reaction, score = 1. If dates on same day check ReactionDesc. 50% penalty if any ambiguity as to whether drug preceded the adverse event. Penalty also applies if drug start date and reaction start date are the same day, and the free‐text does not state that the drug preceded the reaction. No penalty if free‐text states reaction followed drug.
Reaction duration	DurationReaction DurationUnitReaction	If reaction duration given, score = 1. 10% penalty if reaction duration cannot be determined from information given.
Dose	DosageDetails	If dosage can be elucidated from free‐text, score = 1. 10% penalty if the daily dose cannot be determined from free‐text field. No penalty for missing vaccine dose/pipette dose.
Occupation of administrator	Occupation	If populated, score = 1. “Unknown” = 30% penalty. “Other” is not penalized
Breed	Breed	If populated, score = 1. “Unknown” = 30% penalty.
Age	Age	If populated, score = 1. “Unknown” = 30% penalty. 10% penalty imposed if age listed as “0.”
Sex	Sex	If populated, score = 1. “Unknown” = 30% penalty.
Neuter status	NeuterStatus	If populated, score = 1. “Unknown” = 30% penalty.
Comments	ReactionDesc	If populated, score = 1. 10% penalty if no information given (i.e., blank, n/a, …, etc.).
Additional comments	ReactionDescAdditional	If populated with relevant information (either clinical note attached OR mentions no relevant clinical history OR mentions this is the first consult for the animal), score = 1. 10% penalty if clinical notes are not attached without reason (valid reasons for not attaching would be this is first consult or no recent history).

To explore how free text clinical notes may improve the quality of AE reports, the completeness assessment was conducted twice for each report. The first assessment included only the information contained within the report fields, whereas the second assessment considered the information contained within the associated free text clinical notes. The two final scores for each report were compared using the Wilcoxon signed rank test, using R (version 4.4.1).

### 2.6 Comparison With Standard Route Submissions to VMD

To explore the completeness of reports submitted via the SAVSNET AE reporting button to those submitted via standard routes, a random selection of reports submitted directly to the VMD from veterinary professionals in practice over the same time period was analyzed using the adapted vigiGrade criteria. Because of differences in database structure, data was not always captured in the same fields as those shown in Table 2, and instead all relevant fields were considered. The proportion of well‐documented reports submitted via SAVSNET was compared to the proportion of well‐documented reports submitted to the VMD via existing routes using a Chi‐squared test (or Fisher's exact test if categories included < 5 reports) in R (version 4.4.1).

## 3 Results

Reports were received during the pilot testing without incident. One pilot site submitted a feedback form indicating that three veterinary surgeons at their site had submitted test reports. The site estimated that it took 2–5 min to submit a report and strongly agreed that the reporting button facilitates reporting to the VMD. The usability of the button was rated as very good, and no further comments about functionality were provided. The site indicated that they were planning to continue using the button.

Sixty AE reports were submitted in the 18 months after launch. One of these reports related to a suture and therefore was excluded from further assessments. Forty‐two practices submitted an AE, and 11 sites submitted more than one report. All reports contained the four minimum reporting criteria (as stipulated by the relevant VGVP module [12]) and therefore were considered to be valid AE reports.

Approximately half of the reports related to dogs (50.9%, *n* = 30/59), 40.7% (*n* = 24) related to cats, and 6.8% (*n* = 4) related to rabbits. One report concerned an AE in a human; however, because of the auto‐population of animal information on the first data capture screen, the fact that the report concerned a human was only discovered by reading the free text input of the reaction description.

### 3.1 Suspect Products

Over half of the reports concerned products administered by a veterinary surgeon (55.9%, *n* = 33), 37.3% (*n* = 22) related to products administered by the owner, and 5.1% (*n* = 3) were administered by a veterinary nurse. One report did not list the occupation of the person administering the product.

A number of reports included more than one suspect product, and therefore the total number of drug‐event pairs was 76. Overall, seven different product groups were included in the reports, as shown in Table 3. Most reports related to products of the immunological class (64.5%, *n* = 49).

**TABLE 3:** Suspect products as mentioned in the submitted adverse event reports, grouped by ATCVet code first level groups.

ATCVet code first level group	Number of products	Percentage of total products
Immunologicals	49	65.5%
Antiparasitic products, insecticides, and repellents	14	18.4%
Systemic hormonal preparations, excl. sex hormones and insulin	5	6.6%
Musculoskeletal system	4	5.3%
Cardiovascular system	1	1.3%
Antiinfectives for systemic use	1	1.3%
Nervous system	1	1.3%
Unknown	1	1.3%

*Note:* These numbers reflect the overall frequency rather than the number of unique products.

### 3.2 Adverse Events

**FIGURE 2:**
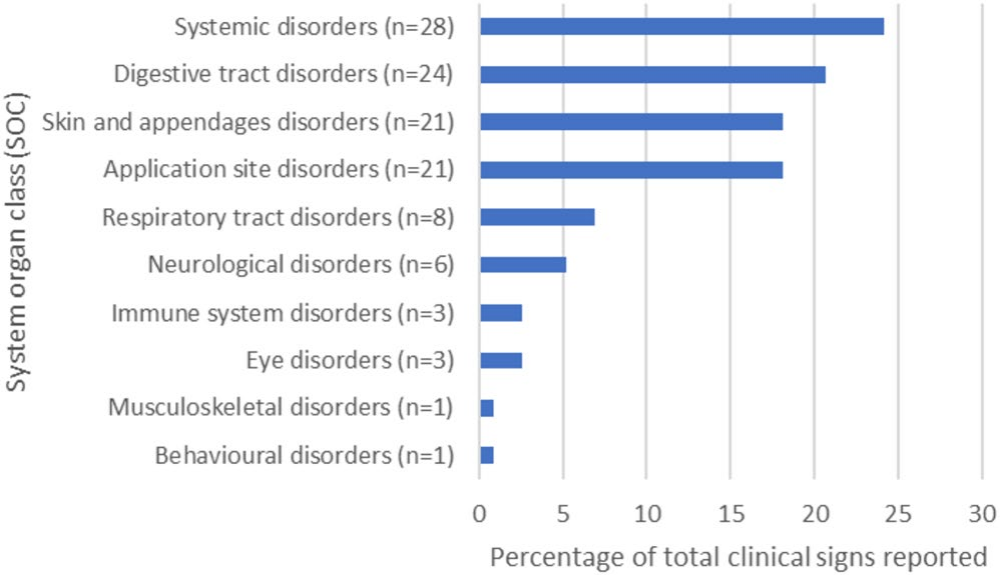
The clinical signs included in the submitted AE reports, grouped by VeDDRA SOC. Note that for some reports multiple clinical signs were reported.

Vomiting and lethargy were the two most commonly reported clinical signs (*n* = 11). In total, 116 clinical signs were reported (63 unique clinical signs), with some reports containing multiple different clinical signs. The AEs reported, grouped by VeDDRA system organ class (SOC) are shown in Figure 2. For the 76 drug‐event pairs, most of these events were considered “expected” (65.8%, *n* = 50/76).

### 3.3 Completeness Assessment

Three assessments were completed using an adapted vigiGrade tool. Firstly, reports received during the first calendar year after launch were assessed. This assessment was repeated, and information contained within the free text clinical notes was also considered as part of the report. Lastly, a sample of reports received by the VMD via standard routes also was assessed.

For the initial assessment, complete agreement was found between reviewers on the scoring of all report elements in 71.1% (*n* = 32/45) of reports. For almost all reports with discrepancies (*n* = 12/13) the disagreement related to scoring of the reaction duration field. After reconciliation, 57.8% (*n* = 26) of reports had scores > 0.80 and therefore were considered well documented. When the reports were re‐assessed to consider the information available within the accompanying free text clinical notes, complete agreement was found between reviewers for 84.4% (*n* = 38) of reports. After reconciliation, 95.6% (*n* = 43) of reports were considered to be well documented.

In the initial assessment, the most common field to receive a penalty was the time‐to‐onset field (*n* = 17/45), followed by the age score (*n* = 14). Notably, no penalties were applied to the time‐to‐onset field in the re‐analysis because this information was available in the clinical notes. However, no further information regarding age was available, and therefore 14 reports still received a penalty for this data element in the second analysis. Completing the vigiGrade assessment of SAVSNET reports using the information contained within the free text clinical notes significantly increased report completeness (*p* < 0.001).

For the reports received by the VMD via standard routes (*n* = 45), complete agreement was found between reviewers for 35.6% (*n* = 16) of reports. For 79.3% (*n* = 23/29) of the reports without complete agreement, the discrepancies related to ascertaining the neuter status of the animal. After reconciliation, 42.2% (*n* = 19) of reports were considered well documented. Descriptive statistics for the three assessments are shown in Table 4. The fields most commonly awarded penalties in the VMD reports were reaction duration (*n* = 26), neuter status (*n* = 23), and dose (*n* = 13).

**TABLE 4:** Descriptive statistics of the completeness analysis conducted using an adapted version of VigiGrade on three datasets: (1) AE reports submitted via SAVSNET utilizing only the information captured within the report fields, (2) AE reports submitted via SAVSNET supplemented with the information available in the free text clinical notes relating to the consultation during which the AE report was submitted, and (3) AE reports submitted directly to the VMD via existing routes.

	SAVSNET AE reports	SAVSNET AE reports and free text	VMD reports
Minimum score	0.20	0.57	0.19
Maximum score	1.00	1.00	1.00
Mean score	0.69	0.94	0.69
Median score	0.81	1	0.63
Interquartile range	0.45	0.1	0.33
Well‐documented reports	57.8% (*n* = 26/45)	95.6% (*n* = 43/45)	42.2% (*n* = 19/45)

Reports submitted via SAVSNET were significantly more likely to be well‐documented than reports submitted to the VMD via standard routes when the assessment included the free text clinical notes (*p* < 0.001). However, this factor was not found to be significant when the information contained within the free text clinical notes was not considered in the assessment (*p* = 0.17).

## 4 Discussion

The reporting of AEs by veterinary professionals forms an important part of the ongoing safety monitoring of veterinary products once they reach the market. Despite this, there is evidence of substantial under‐reporting [4, 5, 6, 7, 8, 9], warranting exploration of methods aimed at increasing the number of AEs reported. In recent years, increasing focus has been placed on the use of electronic reporting tools to improve the reporting rate of AEs in human medicine [16]. Despite surveys suggesting that veterinary professionals perceive the ability to report AEs directly from the PMS as a facilitator to reporting [4, 5, 10], this area remains largely unexplored in veterinary medicine. We utilized the existing infrastructure of SAVSNET to launch an AE reporting functionality onto the main presenting complaint window (which was therefore available during the daily clinical workflow) and used a measure of completeness to assess the quality of reports submitted via this route.

Over the course of 18 months, 60 reports were submitted using the SAVSNET reporting button, and the button was utilized by 11 practices on more than one occasion. As per SAVSNET ethical approval, identifying information is not captured, and therefore it was not possible to ascertain whether reports from these practices were submitted by different individuals working at the practice or if reports were from a single person. Similarly, it was not possible to measure if these practices submitted more reports after introduction of the button because the VMD does not always receive reporter details, especially for reports received via marketing authorization holders.

Given previously indicated support for PMS‐based reporting [4, 5, 10], the proposed rate of AEs [9], and that the reporting functionality was available to 236 practice sites, the number of reports submitted was not as high as expected. One potential explanation for this result is the impact of COVID‐19 on veterinary practice. The reporting functionality was made available to practices during the peak of the COVID‐19 pandemic when restrictions had been re‐introduced in the United Kingdom. During this period, changes in the working practices of veterinary professionals were made to minimize the risk of infection. These included conducting consultations remotely or outdoors to minimize client contact. Given these new and restrictive ways of working, it is understandable that engagement with activities outside of immediate clinical practice decreased. This change is reflected in the AE reporting figures published by the VMD which show a decrease in AE reporting during 2020 [3]. At this time, the VMD has not published their annual pharmacovigilance report for 2021 and so it is not possible to determine if this trend continued through later phases of the pandemic.

Furthermore, a systematic review in human healthcare identified that combining active and passive facilitators of reporting has greater impact on improving reporting rates compared to single intervention approaches [16]. Hence, implementing the reporting functionality in conjunction with a more active facilitator might have further increased its utilization. In human healthcare, appointing a responsible person for AE reporting has been shown to be a successful approach. The introduction of a so‐called adverse drug event manager at a hospital in Denmark [17] and junior adverse drug event managers (i.e., medical students) at a hospital in the Netherlands [18] resulted in large increases in the number of reports submitted over the study periods. In each of these studies, the administrative burden on clinicians was decreased. Similarly, appointing pharmacists as “champions” of AE reporting at hospitals in Wales resulted in an 81% increase of reports submitted when compared to the year before their appointment [19]. Veterinary nurses may be ideal candidates to take on the role of designated reporters. First, in Sweden it has been suggested that veterinary nurses may be aware of AEs that have not been reported and are therefore in a good position to submit AE reports [4]. In addition, veterinary nurses have previously reported that they wish to engage in further pharmacovigilance training and (on a self‐reported basis) have more time to submit AE reports than veterinary surgeons [10]. Veterinary teams however are likely to be smaller thanhuman healthcare teams and there may not always be flexibility or capacity within the team to delegate this task to someone else.

Here, we utilized the vigiGrade assessment to document completeness of the submitted reports and carried out a comparison with reports submitted via standard routes. We adapted the vigiGrade assessment to include important elements relevant to veterinary medicine including breed and neuter status. Although reviewer agreement was initially good when scoring SAVSNET AE reports, agreement was improved further in the second analysis, which included the additional information contained within the free text clinical notes. Notably, agreement was lower for the reports submitted to VMD by standard routes. However, the majority of the discrepancies related to the neuter status of the animal. In the VMD data, sex and neuter status were reported as a single field (i.e., “male‐neutered”). For some animals, only the sex was present, which one of the reviewers interpreted as meaning the animal was not neutered, whereas the second reviewer interpreted this entry as missing information and applied a penalty. This example highlights the importance of having a thorough understanding of the structure of the data being assessed before applying assessment criteria. Furthermore, a large period of time elapsed between assessing the SAVSNET AE reports and assessing the VMD reports, which may have contributed to low reviewer agreement. We suggest when utilizing an assessment such as the one described here that all reviewers be trained and have ample opportunity to practice using the tool before conducting the assessments. Notably, removing the penalty from VMD reports concerning animals where only sex was listed (i.e., considering the lack of comment on neuter status to correspond to an intact animal) improved the number of reports considered to be well‐documented, and therefore did not impact on the overall findings of our study.

Over half of SAVSNET reports were considered to be well‐documented. Furthermore, when reports were supplemented with the information contained within the free text clinical notes, almost all were considered to be well‐documented and these reports were more likely to be classified as well‐documented compared to reports submitted to the VMD via existing routes. The availability of this supplemental information is a considerable advantage of utilizing the PMS for AE reporting. This conclusion is further supported by the fact that all of the penalties initially applied to the SAVSNET reports for the time‐to‐onset field could be resolved by utilizing the information contained within the clinical notes. Should a reporting tool, such as the one described here, be adopted on a larger scale, regulatory bodies would need to consider if the improvement in quality justifies the additional time required to review accompanying clinical notes.

Several SAVSNET reports received a penalty for the age field, which could not be reconciled utilizing information in the clinical notes. All of these penalties were applied due to an age of “0” being populated in this field automatically for animals under one year of age. We suggest that the system be updated to ensure that for animals under one year old the age is populated in months.

Among VMD reports, penalties were most common for the neuter status and reaction duration fields. As previously described, the sex and neuter status fields were combined on VMD reports, and so the penalties applied here could relate to differences in data structure rather than truly missing data. In addition to information regarding the duration of the reaction, the VMD data also contained a data field that captured whether the reaction was ongoing. According to this field, many of the reports with a missing reaction duration were considered ongoing (*n* = 15/26). Because the SAVSNET report form did not have an option to indicate if the reaction was ongoing, direct comparisons of data completeness relating to reaction duration were difficult. Removing the penalty applied to the 15 reports that were reported as ongoing did not increase the number of reports considered to be well‐documented and therefore did not impact the overall findings.

We found that the majority of reports submitted via SAVSNET were 'expected' events (i.e., those already listed in the product literature). Given that an AE being well‐known previously has been shown to be a barrier to reporting [5, 10], this finding is surprising, but most likely reflects the base incidence rate. Adverse event reports concerning known events are important for continued assessment of the frequency of such events to ensure that product information remains accurate. Providing a faster, more efficient route may facilitate report submission and therefore increase the number of AE reports, including expected events, that are submitted.

The main limitation of our study is that the roll‐out of the reporting functionality was constrained to practices participating in SAVSNET that were users of one specific PMS. Participation in SAVSNET is associated with RCVS Practice Standard Scheme points, and therefore these practices may be more likely to engage in activities such as AE reporting. However, given that the number of reports submitted was lower than expected, it is unlikely to have impacted our results. The low number of reports submitted also represents a limitation of the analysis performed. However, given that many elements of the report are pre‐populated from the EHR, even with a larger sample size, reports are likely to be well‐documented. As described, this reporting functionality was developed with efficiency in mind, and therefore several elements of the report pre‐populate based on data in the animal's EHR. A limitation of this approach is that reports relating to humans may not be captured accurately. In future iterations, this problem could be addressed by the addition of a tick box to indicate that the report relates to a human, in which case the animal details would be removed.

## 5 Conclusion and Future Directions

Our study outlines that AE reporting can be integrated into the clinical workflow, and design considerations such as mandatory inclusion of the four minimum reporting criteria, and pre‐population of information from the EHR, along with the availability of additional information contained in the free text clinical notes, results in reports that are at least as well documented as those submitted via standard routes. Although there was a lower than expected uptake during the first 18 months, combining this functionality with another intervention might increase its use. In the future, we intend to explore the utility of appointing so‐called “reporting champions” at individual practices and investigate the impact of doing so on the number of reports submitted using the reporting button. Lastly, consideration should be given to expanding the availability of this functionality to other PMSs, including those not currently compatible with SAVSNET. Broader adoption is likely to require formalized standards within regulatory guidelines or professional schemes to encourage development by PMS providers.

## Disclosure

Authors declare no off‐label use of antimicrobials.

## Ethics Statement

Approved by the University of Liverpool Research Ethics Committee (RETH001081). Authors declare human ethics approval was not needed.

## Conflicts of Interest

Small Animal Veterinary Surveillance Network occasionally receives funding from pharmaceutical companies to conduct specific projects. However, the authors declare that this research was conducted without any commercial or financial relationships that could be construed as a potential conflicts of interest. David Killick is a member of the Veterinary Products Committee, UK. Munir Pirmohamed currently receives partnership funding, paid to the University of Liverpool, for the following: Medicines Research Council (MRC) Clinical Pharmacology Training Scheme (co‐funded by MRC and Roche, UCB, Eli Lilly and Novartis), and the MRC Medicines Development Fellowship Scheme (co‐funded by MRC and GSK, AstraZeneca (AZ), Optum and Hammersmith Medicines Research). He has developed an HLA genotyping panel with MC Diagnostics but does not benefit financially from this arrangement. He is part of the Innovative Medicines Initiative (IMI) Consortium ARDAT (www.ardat.org). The other authors declare no conflicts of interest.
